# Supplementary material for: Sea ice controls net ocean uptake of carbon dioxide by regulating wintertime stratification
Source: Commun Earth Environ. 2025 Jun 18;6(1):457. doi: 10.1038/s43247-025-02395-x (PMC12176633; doi:10.1038/s43247-025-02395-x)
Supplement: Supplementary file 2 — Reporting Summary [file 43247_2025_2395_MOESM2_ESM.pdf]

nature portfolio

Corresponding author(s): Elise S. Droste

Last updated by author(s): May 13, 2025

Reporting Summary

Nature Portfolio wishes to improve the reproducibility of the work that we publish. This form provides structure for consistency and transparency in reporting. For further information on Nature Portfolio policies, see our Editorial Policies and the Editorial Policy Checklist.

Statistics

For all statistical analyses, confirm that the following items are present in the figure legend, table legend, main text, or Methods section.

n/a	Confirmed	
☐	☒	The exact sample size (*n*) for each experimental group/condition, given as a discrete number and unit of measurement
☐	☒	A statement on whether measurements were taken from distinct samples or whether the same sample was measured repeatedly
☒	☐	The statistical test(s) used AND whether they are one- or two-sided<br>*Only common tests should be described solely by name; describe more complex techniques in the Methods section.*
☒	☐	A description of all covariates tested
☒	☐	A description of any assumptions or corrections, such as tests of normality and adjustment for multiple comparisons
☐	☒	A full description of the statistical parameters including central tendency (e.g. means) or other basic estimates (e.g. regression coefficient) AND variation (e.g. standard deviation) or associated estimates of uncertainty (e.g. confidence intervals)
☐	☒	For null hypothesis testing, the test statistic (e.g. *F*, *t*, *r*) with confidence intervals, effect sizes, degrees of freedom and *P* value noted<br>*Give P values as exact values whenever suitable.*
☒	☐	For Bayesian analysis, information on the choice of priors and Markov chain Monte Carlo settings
☒	☐	For hierarchical and complex designs, identification of the appropriate level for tests and full reporting of outcomes
☒	☐	Estimates of effect sizes (e.g. Cohen's *d*, Pearson's *r*), indicating how they were calculated

Our web collection on statistics for biologists contains articles on many of the points above.

Software and code

Policy information about availability of computer code

Data collection	No software was used in data collection.
Data analysis	Calkulate Python package (version 23.2.2; Humphreys et al., 2022a) was used to determine the total alkalinity values in units micro mole per kilogram from the raw data of the potentiometric titration method. PyCO2SYS Python package (version 1.8; Humphreys et al., 2022b) was used to determine the fugacity of CO2 based on the dissolved inorganic carbon, total alkalinity, salinity, seawater temperature, and nutrient data.

For manuscripts utilizing custom algorithms or software that are central to the research but not yet described in published literature, software must be made available to editors and reviewers. We strongly encourage code deposition in a community repository (e.g. GitHub). See the Nature Portfolio guidelines for submitting code & software for further information.

Data

Policy information about availability of data

All manuscripts must include a data availability statement. This statement should provide the following information, where applicable:

- Accession codes, unique identifiers, or web links for publicly available datasets
- A description of any restrictions on data availability
- For clinical datasets or third party data, please ensure that the statement adheres to our policy

Access details for Rothera Time Series data can be found in Venables et al. (2023). Rothera Time Series DIC and TA data between 2010-2014 has been made

available on the Ocean Carbon and Acidification Data System (OCADS; Legge et al., 2017). DIC and TA data between 2014-2020 will be made publicly available on the UK Polar Data Centre before publication of the manuscript.

Research involving human participants, their data, or biological material

Policy information about studies with human participants or human data. See also policy information about sex, gender (identity/presentation), and sexual orientation and race, ethnicity and racism.

Reporting on sex and gender	N/A
Reporting on race, ethnicity, or other socially relevant groupings	N/A
Population characteristics	N/A
Recruitment	N/A
Ethics oversight	N/A

Note that full information on the approval of the study protocol must also be provided in the manuscript.

Field-specific reporting

Please select the one below that is the best fit for your research. If you are not sure, read the appropriate sections before making your selection.

☐ Life sciences ☐ Behavioural & social sciences ☒ Ecological, evolutionary & environmental sciences

For a reference copy of the document with all sections, see nature.com/documents/nr-reporting-summary-flat.pdf

Ecological, evolutionary & environmental sciences study design

All studies must disclose on these points even when the disclosure is negative.

Study description	This study uses observational, in-situ data collected in Ryder Bay, along the West Antarctica Peninsula, to study the interannual variability of the surface marine carbonate system, as part of the Rothera Time Series (RaTS). The purpose of seawater samples collected for this study is to quantify the dissolved inorganic carbon (DIC) content and total alkalinity (TA) content in the surface marine layer. Interpretation of the results is done using results of the wider and longer-term RaTS variable array, including seawater temperature, salinity, nutrients, seawater oxygen isotopes, and sea ice observations. In particular, we focus on the role of seasonal sea ice (and its interannual variability) on the water column physical properties and carbonate chemistry (including fugacity of CO2), and how that explains interannual variability in the surface marine carbonate system and sea-air CO2 exchange. Following previous work that showed a clear relationship between annual sea ice cover duration and water column stratification (in winter, but also in the following spring/summer), we categorised data from different years in the carbonate chemistry time series as either low stratification years or high stratification years (associated to short sea ice cover and long sea ice cover duration, respectively), based on a mean wintertime stratification threshold. We then compared the mean seasonal evolution between these two categories for DIC, TA, salinity, as well as derived variables, such as fugacity of CO2 (fCO2) and the contribution of deep water to the surface layer due to mixing.
Research sample	The research sample is seawater collected from the marine surface layer, which is analysed for dissolved inorganic carbon (DIC) and total alkalinity (TA). These samples are collected year-round in Ryder Bay, along the West Antarctic Peninsula, as part of the Rothera Time Series (RaTS), which aims to understand drivers behind seasonal and interannual variability of the marine biogeochemical and physical system in this Antarctic coastal region. To understand the marine carbonate system, DIC/TA were added in 2010 to the variable array of the RaTS, which started in 1998. To accurately quantify all components of the marine carbonate system, including fCO2, we need measurements of two marine carbonate system variables. Advantages of measuring DIC and TA (instead of fCO2 and/or pH) include lower chance of errors introduced due to temperature and pressure changes, and practical sampling conditions as it does not require much specialised equipment in the field. DIC and TA measurements allow us to characterise the marine carbonate system and asses the role of biogeochemical processes. The data of RaTS are considered representative for Ryder Bay, and likely also the larger adjacent Marguerite Bay. While the spatial scale of RaTS data is small, the motivation behind RaTS is to gain a mechanistic understanding of processes behind seasonal- and interannual variability. This is an ongoing study with a growing temporal spread, but the dataset is now considered to contain sufficient data and years, as well as sufficient interannual variability, to study the effects of varying sea ice conditions and vertical ocean mixing on the surface marine carbonate system.
Sampling strategy	Seawater samples for DIC/TA analysis were collected from December 2010 onwards at 15 m depth to remain consistent with the sampling strategy of the Rothera Time Series, which started in 1998 (i.e., for Conductivity-Temperature-Depth casts and nutrient samples). In 1998, 15 m was chosen as the primary seawater sampling depth of the time series, as it is the mean depth of the chlorophyll maximum. Samples were collected from the side of a small boat or through a hole in the ice, following Standard Operation Procedure 1 in Dickson et al. (2007), which avoids contamination of CO2 by minimising contact of the sample to the atmosphere. Depending on weather and ice conditions, sampling is done either weekly or every two weeks, which is considered often enough to capture seasonal and interannual variability of the marine system. Samples were fixed in the lab at Rothera Research station (SOP1), to stop biological processes altering the carbonate chemistry of the sample.

Data collection	DIC/TA samples were shipped by the British Antarctic Survey to the UK roughly every year, where they were analysed on a VINDTA 3C system at the University of East Anglia, Norwich. Exceptions are samples collected between 2013 and 2016, which were analysed onsite at Rothera Research station, using the same analytical techniques: coulometric titration for DIC and potentiometric titration for TA. All raw analytical data were computationally processed following Standard Operation Procedures in Dickson et al. (2007). This was done by Oliver Legge for data between 2010 and 2014 (Legge et al., 2015), by Elizabeth Jones for data between 2014-2016, and by Elise Droste for data between 2016-2020. Metadata and sea ice observations were collected by the Marine Scientists at Rothera Research station.
Timing and spatial scale	Collection of DIC/TA samples started on 28 December 2010. Sampling is still ongoing, but the latest sample included in our study was collected on 28 February 2020. Samples are collected year-round, including in winter. Frequency of sampling is weekly in most parts of the year, or whenever weather and ice conditions allowed. Weekly sampling or sampling every two weeks is considered often enough to capture seasonal and interannual variability of the marine system. However, sampling frequency is highly dependent on weather and sea ice conditions and the number of available Marine Scientists on base at Rothera (i.e., normally two Marine Scientists in summer and one in winter, as this is fitting for the time commitments of the role). Sampling frequency is therefore often reduced to roughly every two weeks in winter. The dataset now has sufficient years with sufficient interannual variability to study the effects of varying sea ice conditions and mixing on the surface marine carbonate system. All samples were collected in Ryder Bay, about 4 km offshore. The vast majority of samples were collected from a single sampling site (i.e., site 1 at 67°34.200'S, 68°13.500'W). In harsh environmental conditions, samples were collected at sites 2 or 3 (i.e., 67°34.850'S, 68°9.340'W and 67°34.330'S, 68°7.970'W, respectively). Previous studies have found that the surface seawater processes at sites 2 and 3 are representative of those occurring at site 1 (Legge et al., 2015).
Data exclusions	Data were excluded from further analysis when they did not pass the quality checking stage when processing the raw dissolved inorganic carbon/total alkalinity (DIC/TA) data from the VINDTA. Results for Certified Reference Materials were excluded when the analytical instrument (VINDTA) clearly did not perform well (usually observed in the lab), or when their reproducibility was poor. Results for seawater samples were excluded if the instrument performed poorly, or when the sample had been compromised due to contamination during sampling and/or storage (e.g., if the sample was not gas-tight).
Reproducibility	Data used in this study was acquired in the field as part of a time series of a marine system. No experiments were performed. Reproducibility was only applicable in the running the Certified Reference Material (CRMs) on the instruments that measure dissolved inorganic carbon and total alkalinity: We analysed at least two CRM bottles from the same batch, on every analysis day, and each bottle was run twice. The results of these runs were used to quality check the instrument, as well as to determine the analytical uncertainty of the measurements.
Randomization	Datapoints were allocated into two groups: "low stratification years" or "high stratification years". Allocation depended on whether the datapoint belonged to a year in which the mean winter stratification was below or above 1500 J m-2. The purpose of categorising the datapoints in this manner was to study mechanisms behind interannual variability in a time series, and therefore randomization was not applicable to this study.
Blinding	The purpose of categorising datapoints into "low stratification years" or "high stratification years" was to study mechanisms behind interannual variability in a time series, and therefore blinding was not applicable to this study.

Did the study involve field work? ☒ Yes ☐ No

Field work, collection and transport

Field conditions	Seawater samples were collected year-round since December 2010, capturing environmental conditions in all seasons. Field conditions thus varied with the seasons. Air temperatures at Rothera Research Station range from -5 °C to -20 °C in winter to 0 °C and +5 °C in summer. Ryder Bay has sea ice from late May to late November. Snow can fall any time of year, but is more common at the end of winter. Rain sometimes falls at Rothera. As Rothera is south of the Antarctic circle, it has 24 hours of daylight in summer and 24 hours of darkness in winter (BAS website, https://www.bas.ac.uk/polar-operations/sites-and-facilities/facility/rothera). Seawater samples were collected from a small boat, which can be navigated through brash ice and break very thin new ice, or from a sledge on ice. For logistical and safety reasons, sampling activities were avoided in harsh conditions (e.g., strong winds > 15 knots, impassable ice, or poor visibility), and thus environmental conditions normally set the frequency/timing in winter.
Location	Seawater samples were collected at 3 sampling sites in Ryder Bay, along the West Antarctic Peninsula: site 1 (67°34.200'S, 68°13.500'W), site 2 (67°34.850'S, 68°9.340'W), and site 3 (67°34.330'S, 68°7.970'W). Respective water depths at sites 1, 2, and 3 are 520 m, ~400 m, and <100 m. Seawater samples used in this study were collected at 15 m depth at all sites. The vast majority of samples were collected at site 1.
Access & import/export	Data collection for the Rothera Time Series is led by the British Antarctic Survey, who have relevant Antarctic Permissions by following the requirements of the Protocol on Environmental Protection to the Antarctic Treaty (1991) and the provisions of the Antarctic Act 1994, the Antarctic Act 2013, and accompanying Antarctic Regulations 1995/490. This includes a Preliminary Environmental Assessment (PEA), which is an environmental impact assessment that includes mitigation measures, such as careful control of contents on the boat to minimise loss of equipment in the sea, following all sampling protocols, following all training on fuel for boat engines and boat driving, especially in the presence of marine mammals. Chemical spills are mitigated by using chemicals to fix the DIC/TA samples only in the lab at the land-based Rothera Research Station and following all on-site procedures for storage and transport of chemicals. Incidents are made known immediately.
Disturbance	Disturbance to the environment and wildlife was minimised by following the strict procedures described in the PEA forms. The boats used during sampling are small and are coasted onto Rothera Research Station to reduce mixing of the water column before CTD casts and water sampling. The CTD cast is deployed first, followed by seawater sampling, to reduce time between water sampling and

lab processing on land.

Reporting for specific materials, systems and methods

We require information from authors about some types of materials, experimental systems and methods used in many studies. Here, indicate whether each material, system or method listed is relevant to your study. If you are not sure if a list item applies to your research, read the appropriate section before selecting a response.

Materials & experimental systems

n/a	Involved in the study
☒	☐ Antibodies
☒	☐ Eukaryotic cell lines
☒	☐ Palaeontology and archaeology
☒	☐ Animals and other organisms
☒	☐ Clinical data
☒	☐ Dual use research of concern
☒	☐ Plants

Methods

n/a	Involved in the study
☒	☐ ChIP-seq
☒	☐ Flow cytometry
☒	☐ MRI-based neuroimaging

Plants

Seed stocks	N/A
Novel plant genotypes	N/A
Authentication	N/A
